# Computation of haplotypes on SNPs subsets: advantage of the "global method"

**DOI:** 10.1186/1471-2156-7-50

**Published:** 2006-10-26

**Authors:** Cédric Coulonges, Olivier Delaneau, Manon Girard, Hervé Do, Ronald Adkins, Jean-Louis Spadoni, Jean-François Zagury

**Affiliations:** 1 Equipe génomique, bioinformatique et pathologies du système immunitaire, INSERM U736, 15 rue de l'École de Médecine, 75006 Paris, France; 2 Chaire de Bioinformatique, Conservatoire National des Arts et Métiers, 292 rue Saint-Martin, 75003 Paris, France; 3 Children's Foundation Research Center and Center of Genomics and Bioinformatics, University of Tennessee, Memphis, TN, USA

## Abstract

**Background:**

Genetic association studies aim at finding correlations between a disease state and genetic variations such as SNPs or combinations of SNPs, termed haplotypes. Some haplotypes have a particular biological meaning such as the ones derived from SNPs located in the promoters, or the ones derived from non synonymous SNPs. All these haplotypes are "subhaplotypes" because they refer only to a part of the SNPs found in the gene. Until now, subhaplotypes were directly computed from the very SNPs chosen to constitute them, without taking into account the rest of the information corresponding to the other SNPs located in the gene. In the present work, we describe an alternative approach, called the "global method", which takes into account all the SNPs known in the region and compare the efficacy of the two "direct" and "global" methods.

**Results:**

We used empirical haplotypes data sets from the *GH1 *promoter and the *APOE *gene, and 10 simulated datasets, and randomly introduced in them missing information (from 0% up to 20%) to compare the 2 methods. For each method, we used the PHASE haplotyping software since it was described to be the best. We showed that the use of the "global method" for subhaplotyping leads always to a better error rate than the classical direct haplotyping. The advantage provided by this alternative method increases with the percentage of missing genotyping data (diminution of the average error rate from 25% to less than 10%). We applied the global method software on the GRIV cohort for AIDS genetic associations and some associations previously identified through direct subhaplotyping were found to be erroneous.

**Conclusion:**

The global method for subhaplotyping can reduce, sometimes dramatically, the error rate on patient resolutions and haplotypes frequencies. One should thus use this method in order to minimise the risk of a false interpretation in genetic studies involving subhaplotypes. In practice the global method is always more efficient than the direct method, but a combination method taking into account the level of missing information in each subject appears to be even more interesting when the level of missing information becomes larger (>10%).

## Background

Large-scale genomic studies are becoming a standard nowadays. The exploitation of this huge body of data leads to multiple biological applications and in particular, to the unraveling of new molecular mechanisms for diseases through the identification of genetic associations. Genetic association studies are based on the comparison of genetic markers, the most frequent ones being Single Nucleotide Polymorphisms (or SNPs), between a diseased group versus a healthy group (case-control study). If a statistically significant difference is observed in the frequency of a SNP allele between a group of patients and a group of control subjects, it could mean that the gene or its product is involved in disease development. Association studies must also be performed on haplotypes which are the combination of SNPs in a given locus. Indeed, haplotypes and not only SNPs have already been reported to be associated with complex diseases such as AIDS [1-4], cancer [5-7], or Alzheimer's disease [8].

Experimental methods for haplotyping exist such as long-range haplotyping [9], single-copy DNA genotyping in conjunction with the Mass ARRAY system [10], or clone-based systematic haplotyping [11] but they are not applicable at a large scale level because of cost and time consumption. As an alternative, computational approaches have been developed to derive haplotypes from the SNP genotypic information (the couple of alleles found for each SNP) in a whole population. The most widely used algorithms to infer haplotypes from the unphased genotypic data rely today on statistical approaches such as the expectation-maximization (EM) algorithm or Bayesian coalescence-based algorithms [12,13].

Haplotypes have been the subject of an increasing number of studies in the recent years. Haplotypes information makes it possible to highlight the structure of the genome, notably through haploblocks which correspond to segments of chromosomes unlikely to undergo a crossing-over event [14,15]. In order to spare repeated efforts, an international consortium has undertaken the HapMap project with the aim of providing an exhaustive map including the most important SNPs determining the most frequent haplotypes in each haploblock of the human genome. The Hapmap project could accelerate the detection of SNP alleles or haplotypes associated with a disease phenotype [11].

The inference of haplotypes by computational methods can be very difficult and even sometimes incorrect. Indeed, the number of candidate haplotypes increases exponentially with the number of polymorphic sites, this number being 2^n ^in a subject with n heterozygous SNPs. Thus, it is not generally possible to solve correctly the equations (infer their haplotypes) for all subjects especially when there are missing data (SNPs whose alleles are unknown for some subjects in the population) which happens in most experiments.

Recent studies have compared the various computational methods to derive haplotypes [16-18]. Among them, the PHASE software [19] seemed to yield better results [13,16,20]. However, when haplotypes involving more than 7 SNPs were estimated from unphased genotypes, the reliability was poor even for PHASE, with an error rate jumping as high as 10%. It can thus become very useful to study haplotypes based on smaller set of SNPs in the population, which we will call here "subhaplotypes", because of the higher degree of experimental reliability (less missing data) and the higher degree of accuracy (for the haplotype computation).

It can also be important to investigate subhaplotypes with regard to their putative biological function: for instance subhaplotypes derived from SNPs in the gene promoter region [21,22], derived from SNPs leading to a protein mutation [21], or derived from tagSNPs [23-26]. Up to now, subhaplotypes derived from a set of selected SNPs in a gene have most often been inferred in a population by using only the genotypic information of these very SNPs in this population. However, an alternative approach could be to estimate the haplotypes from all the SNPs found in the gene and then, from these large haplotypes, extract the subhaplotypes corresponding to the set of the selected SNPs. In the case of missing information among the SNPs this approach might be useful because the missing information can be compensated through the linkage disequilibrium existing with other SNPs in the gene [27]. The first method, based on the direct haplotyping of SNPs of interest, will be called the "direct method". The second method, based on the use of larger haplotypes (haplotypes containing a larger number of SNPs) to infer subhaplotypes will be called the "global method".

The purpose of the present study is to evaluate which subhaplotyping procedure was optimal by comparing them on real and artificial genomic datasets. Such a comparative evaluation has not been performed before and it is particularly important for two reasons: 1. up to now most reports on disease genetic association studies use the "direct method" to estimate subhaplotypes [22,28-30]. 2. Many groups focus only on a limited set of representative SNPs such as tagSNPs to compute haplotypes [31,32] when they could use a larger set of SNPs to compute haplotypes more accurately.

## Results

The goal of this study is to compare the two subhaplotyping strategies, "direct" and "global". For the comparison of the two strategies, we have first used two real haplotype datasets previously determined experimentally: haplotypes determined experimentally on 150 Caucasian subjects in the *GH1 *gene and corresponding to 14 SNPs with a MAF>1%, and haplotypes data determined experimentally on 80 subjects of various ethnical backgrounds in the *APOE *gene and corresponding to 9 SNPs with a MAF>1%. These experimentally determined haplotypes have been previously used as test samples by other researchers [16,33,34]. We have also used 10 simulated haplotype datasets artificially generated using a coalescent model on 30 SNPs and 100 individuals using the method of Schaffner et al. [35]. All these datasets are described in more details in Material and Methods. In order to look like real genomic data we have also introduced artificially missing information at various rates (2%, 5%, 10%, 15%, and 20%) in these datasets (see Material and Methods).

For the 2 methods, the computation of estimated haplotypes was done with the PHASE software, previously shown to be more reliable than the other haplotyping software [13,16,20,36]. The comparison of the 2 subhaplotyping methods, "direct" and "global", was performed with the following coefficients: the individual error rate for haplotype assignment (the 2 haplotypes assigned to an individual were correct or not), the similarity error rate [13] which measures the number of mutations required to obtain the real haplotypes for an individual, and the I_F _coefficient which compares the estimated haplotype frequencies with the real ones [37]. All these coefficients are extensively described in Material and Methods.

Finally, we compared the impact of the use of the "global" and the "direct" methods in real genomic data obtained from an AIDS case-control study, the GRIV study, which compares extreme profiles of progression to AIDS with seronegative controls [38].

### Comparison of the 2 methods in the GH1 haplotypes dataset

We tested various SNPs subsets of the *GH1 *data set to do the comparison of the 2 subhaplotyping methods: we first randomly generated 100 subsets with no missing data for each size of 3, 5, and 7 SNPs. We then created randomly missing data in the genotypic dataset at various rates of 2%, 5%, 10%, 15%, and 20%, 20 times for each rate (a total of 100 genotypic datasets) and then, for each dataset, we generated randomly 20 subsets for each size of 3, 5, and 7 SNPs to compare the 2 methods after introducing missing data (see Material and Methods). Overall, for a given size of SNP subset (3, 5, or 7 out of the 14 SNPs) we tested 100 samples with no missing information, and 2000 samples with missing information.

**Figure 1:**
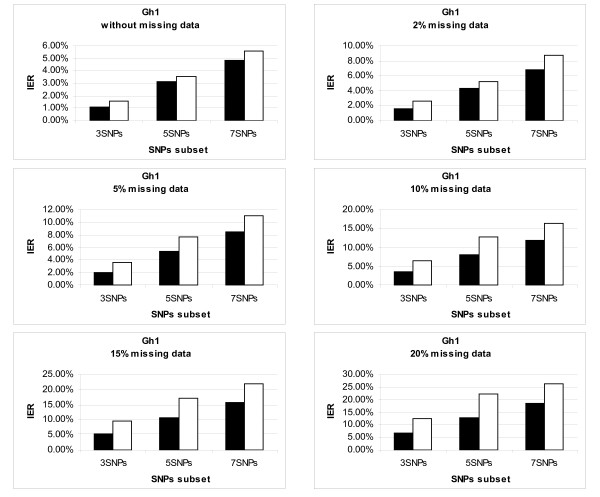
**Graphical representation comparing the individual error rates (IER) between the direct and global methods**. This figure presents the detailed graphs of the average error rates obtained by the 2 subhaplotyping methods, "direct" (in white) and "global" (in black), when they rely on the resolution with maximum probability (Rmax) produced by PHASE. Each graph corresponds to a different level of missing data introduced in the GH1 genotypic dataset (0%, 2%, 5%, 10%, 15% and 20%) and presents the mean of IER of all the replicates tested. There error rate obtained by the global method is always lower.

We compared the global and direct methods on the measure "maximal resolution" (Rmax) corresponding to the haplotypes with the highest probability assigned by PHASE. Interestingly, we noticed in these tests that PHASE always managed to determine at least one possible resolution for each patient. Figure 1 shows graphs giving the mean individual error rate (IER) of both methods according to the rate of missing information. One may observe that the global method appeared to systematically yield a smaller mean error than the direct method (Figure 1). Also, it was not surprising to observe that the level of error of subhaplotype estimates produced by both methods increased with the number of SNPs involved for subhaplotyping and with the level of missing information: a range of 1 to 5% errors with no missing genotypic information to a range of 5 to 25% errors with 20% of missing genotypic information (Figure 1).

Table 1 further analyzes the difference between the 2 methods by presenting the similarity error rate (SimER) and the I_F _coefficients (see Material and Methods): the global method clearly yields better results.

**Table 1:** Error rates obtained according to the level of missing information in the GH1 dataset

*GH1*					
MD	Method	IER	SimER	I_F_	Res rate

0%	**Global_3snp**	**1.12%**	**0.37%**	**0.994**	**100%**
	Local_3snp	1.56%	0.52%	0.9904	100%
	**Global_5snp**	**3.16%**	**0.65%**	**0.9834**	**100%**
	Local_5snp	3.57%	0.72%	0.9784	100%
	**Global_7snp**	**4.87%**	**0.74%**	**0.9714**	**100%**
	Local_7snp	5.57%	0.83%	0.9704	100%
					
2%	**Global_3snp**	**1.63%**	**0.38%**	**0.9937**	**100%**
	Local_3snp	2.57%	0.68%	0.9898	100%
	**Global_5snp**	**4.34%**	**0.88%**	**0.9826**	**100%**
	Local_5snp	5.26%	1.20%	0.9792	100%
	**Global_7snp**	**6.83%**	**0.82%**	**0.9693**	**100%**
	Local_7snp	8.70%	1.03%	0.961	100%
					
5%	**Global_3snp**	**2.03%**	**0.46%**	**0.9934**	**100%**
	Local_3snp	3.65%	0.79%	0.9894	100%
	**Global_5snp**	**5.40%**	**0.88%**	**0.98**	**100%**
	Local_5snp	7.68%	1.20%	0.972	100%
	**Global_7snp**	**8.43%**	**1.07%**	**0.967**	**100%**
	Local_7snp	11.00%	1.37%	0.959	100%
					
10%	**Global_3snp**	**3.57%**	**0.73%**	**0.989**	**100%**
	Local_3snp	6.60%	1.33%	0.983	99.87%
	**Global_5snp**	**8.06%**	**1.19%**	**0.973**	**100%**
	Local_5snp	12.91%	1.86%	0.962	100%
	**Global_7snp**	**11.84%**	**1.35%**	**0.959**	**100%**
	Local_7snp	16.41%	1.88%	0.946	100%
					
15%	**Global_3snp**	**5.21%**	**1.01%**	**0.987**	**100%**
	Local_3snp	9.49%	1.84%	0.977	99.45%
	**Global_5snp**	**10.67%**	**1.47%**	**0.97**	**100%**
	Local_5snp	17.00%	2.33%	0.953	100%
	**Global_7snp**	**15.70%**	**1.67%**	**0.953**	**100%**
	Local_7snp	21.87%	2.35%	0.931	100%
					
20%	**Global_3snp**	**6.65%**	**1.26%**	**0.984**	**98.44%**
	Local_3snp	12.45%	2.41%	0.97	97.02%
	**Global_5snp**	**12.83%**	**1.72%**	**0.964**	**100%**
	Local_5snp	22.15%	3.02%	0.936	99.60%
	**Global_7snp**	**18.47%**	**1.92%**	**0.946**	**99.72%**
	Local_7snp	26.44%	2.81%	0.916	100%

Summary of the mean error rates obtained by each subhaplotyping method when they used the Rmax resolution produced by PHASE. The average individual error rate (IER) and similarity error rate (SimER) were computed according to the level of missing data (MD) introduced in the population (0%, 2%, 5%, 10%, 15%, 20%). Tests were performed on randomly selected SNP subsets of size 3, 5, and 7 taken out of the 14 SNPs present in the *GH1 *genomic dataset (see text and Material and Methods). This table presents the average of the I_F _coefficients which compares the accuracy of the subhaplotypes frequencies found by each subhaplotyping method. The global method fares always better.

### Comparison of the 2 methods in other haplotypes datasets

We analyzed in the same way another real haplotype dataset, previously published by Orzack et al. [33]. As shown in Table 2, the global method again yields better results. We also generated a population with artificial haplotypes as described in Schaffner et al. [35], and found similarly that the global method was more accurate (Table 2).

**Table 2:** Error rates obtained according to the level of missing information in the APOE and simulated datasets

*APOE*					
MD	Method	IER	SimER	I_F_	Res rate

0%	**Global**	**1.94%**	**0.45%**	**0.986**	**100%**
	Local	4.88%	1.22%	0.97	100%
2%	**Global**	**2.24%**	**0.48%**	**0.986**	**100%**
	Local	5.12%	1.19%	0.972	100%
5%	**Global**	**3.20%**	**0.54%**	**0.987**	**100%**
	Local	5.64%	1.83%	0.978	100%
10%	**Global**	**4.41%**	**0.68%**	**0.979**	**100%**
	Local	6.89%	1.91%	0.972	100%
15%	**Global**	**6.98%**	**1.09%**	**0.974**	**100%**
	Local	10.33%	1.97%	0.964	99.75%
20%	**Global**	**12.35%**	**2.09%**	**0.954**	**100%**
	Local	15.21%	2.54%	0.943	99.21%
					
*Simulated*					

MD	Method	IER	SimER	I_F_	Res rate

0%	**Global**	**0.14%**	**0.02%**	**0.996**	**100%**
	Local	0.81%	0.10%	0.989	100%
2%	**Global**	**0.19%**	**0.02%**	**0.989**	**100%**
	Local	1.26%	0.13%	0.982	100%
5%	**Global**	**0.25%**	**0.03%**	**0.982**	**100%**
	Local	1.46%	0.18%	0.975	100%
10%	**Global**	**0.46%**	**0.05%**	**0.968**	**100%**
	Local	2.65%	0.32%	0.961	100%
15%	**Global**	**0.83%**	**0.09%**	**0.954**	**100%**
	Local	4.80%	0.59%	0.947	100%
20%	**Global**	**1.51%**	**0.16%**	**0.941**	**100%**
	Local	8.70%	1.06%	0.934	100%

Summary of the average error rates found when working with subhaplotypes based on 4 SNPs out of 9 in the *APOE *genomic dataset and 10 SNPs out of 30 in the 10 simulated datasets.

IER: individual error rate, Res Rate: resolution rate, SimER: similarity error rate, MD: Missing data, I_F_: frequency error rate (see Material and Methods).

Interestingly, one can see that if the global method is always better, the values of the IER, SER, and I_F _coefficients obtained by each method are different between the GH1, ApoE and artificial datasets for each level of missing information (see Table 1 and 2). The genetic structure of the population at stake appears thus to be very important.

### Statistical significance

The results shown in Table 1 give the mean values of the error levels, however it does not give the number of times when the global method gets an error level lower than the direct method. We did this computation and found for the GH1 gene that the global method provided a more accurate result in 87% of the tests with no missing information, in 88% of the tests with 2% missing information, in 90% of the tests with 5% missing information, in 92% of the tests with 10% missing information, in 95% of the tests with 15% missing information, in 97% of the tests with 20% missing information. Similar results were obtained for APOE and the simulated SNP data (data not shown).

We also performed ANOVA tests to compare the IER obtained by both methods on each subset of a given size (GH1_3SNPs, GH1_5SNPs, GH1_7SNPs, APOE_4SNPs, SIM1 to SIM10_10 SNPs). The results (data not shown) show again that the IER obtained by the global method are significantly better (p < 10^-4^) than the IER found by the direct method for all subsets of SNPs.

### Use of haplotypes defined through a Rmax cut-off

Since biologists often prefer to work with very clean data, we decided to select the most likely resolutions produced by PHASE. We thus selected those resolutions which exhibited an output probability higher than either 50% or 70% (see Material and Methods). Table 3 shows the results obtained by the direct and the global methods. For both the 50% and the 70% cut-offs, the global method yielded an error rate similar to the local method but it also yielded many more resolutions (Table 3). The global method with a 50% cut-off led to slightly more errors than the global method at 70% cut-off, but it also yielded many more resolutions (Table 3). Finally, when one compares the results obtained with cut-offs (Table 3) with the results obtained by Rmax (Table 1), it seems that the number of resolutions obtained by Rmax (it is always 100%) is higher than the number of resolutions obtained when using a cut-off, however the error rate is not as much different. In other words, the use of cut-offs leads to more accurate resolutions but a smaller percentage of patients gets subhaplotyped.

**Table 3:** Error rates found by each method when using cut-offs for the probabilities provided by PHASE

*GH1 – cutoff 70% (5 SNPs)*				
MD	**Method**	Abs IER	Rel IER	Res rate

0%	**Global**	**2.18%**	**2.24%**	**97.31%**
	Local	2.56%	2.65%	96.54%
2%	**Global**	**2.92%**	**2.99%**	**96.78%**
	Local	2.97%	3.05%	94.78%
5%	**Global**	**3.33%**	**3.49%**	**95.38%**
	Local	3.17%	3.51%	90.42%
10%	**Global**	**4.50%**	**4.92%**	**91.40%**
	Local	4.07%	5.00%	81.50%
15%	Global	5.83%	6.56%	88.88%
	**Local**	**4.85%**	**6.42%**	**75.54%**
20%	**Global**	**7.06%**	**8.14%**	**86.80%**
	Local	6.00%	8.81%	68.12%
				
*GH1 – cutoff 50% (5 SNPs)*				

MD	**Method**	Abs IER	Rel IER	Res rate

0%	**Global**	**2.99%**	**2.99%**	**99.82%**
	Local	3.50%	3.50%	99.83%
2%	**Global**	**3.82%**	**3.98%**	**99.81%**
	Local	4.18%	4.26%	97.47%
5%	**Global**	**5.03%**	**5.06%**	**99.42%**
	Local	5.08%	5.32%	95.45%
10%	**Global**	**7.17%**	**7.28%**	**98.45%**
	Local	7.24%	8.04%	90.06%
15%	**Global**	**9.41%**	**9.62%**	**97.84%**
	Local	9.50%	10.95%	86.83%
20%	**Global**	**11.25%**	**11.58%**	**97.18%**
	Local	11.96%	14.45%	82.77%

This table presents the summary of average error rates when using a cut-off on resolution probability given by PHASE instead of Rmax when choosing the resolution obtained by each method. The 2 cut-offs chosen were respectively 50% and 70%. The error rate is almost always lower than with Rmax, but the number of assigned haplotypes strongly decreased.

IER: individual error rate, Abs IER: absolute IER, Rel IER: relative IER, Res Rate: resolution rate, MD: Missing Data.

### Combinations of the global method with the direct method according to the relative percentage of missing data

We reasoned that the localization of the missing information in each patient could influence the output on the global method versus that of the direct method. We thus tried a last approach to optimize the quality of the results: combining the global and direct methods when their results for the most probable resolution (Rmax) are different for a given patient (discordant subhaplotypes). For a patient, if the missing information rate was higher among the very SNPs selected for subhaplotyping, the subhaplotype provided by the global method was chosen; otherwise the subhaplotype provided by the direct method was chosen (see Material and Methods).

As shown in Table 4, the combination method gave a rather good rate of error compared with the global method but there were slightly less patients' haplotypes resolved. Its use appeared most valuable when the number of missing information was higher than 15% (Table 4): the rate of error kept low (less than 7%), while the number of resolved patients remained high (around 90%). The application of this method on the *APOE *gene and on simulated data yielded similar results and conclusions (data not shown).

**Table 4:** Error rates of the combination method

*Combination*					
MD	Method	Abs IER	Rel IER	SimER	Res rate

2%	combi_3snp	1.83%	1.84%	0.17%	100.00%
	combi_5snp	2.56%	2.57%	0.36%	99.58%
	combi_7snp	4.89%	4.89%	0.54%	99.23%
					
5%	combi_3snp	2.72%	2.72%	0.29%	100.00%
	combi_5snp	3.64%	3.69%	0.57%	98.39%
	combi_7snp	6.09%	6.27%	0.60%	97.15%
					
10%	combi_3snp	3.16%	3.22%	0.45%	98.06%
	combi_5snp	5.60%	5.81%	0.68%	96.40%
	combi_7snp	8.12%	8.54%	0.67%	95.12%
					
15%	combi_3snp	5.03%	5.09%	0.61%	98.81%
	combi_5snp	5.98%	6.31%	0.82%	94.70%
	combi_7snp	6.62%	7.36%	0.77%	89.95%
					
20%	combi_3snp	4.09%	4.56%	0.83%	89.78%
	combi_5snp	8.16%	8.70%	0.96%	93.80%
	combi_7snp	6.43%	7.34%	0.90%	87.54%

This table presents the summary of average error rates when using the combination method (see Material and Methods). In that case, we present the example of the results obtained for subsets of 5 SNPs.

IER: individual error rate, Abs IER: absolute IER, Rel IER: relative IER, SimER: similarity error rate, Res Rate: resolution rate, MD: Missing Data.

### Application to the analysis of subhaplotypes in an AIDS cohort

GRIV (Genetics of Resistance to immunodeficiency Virus) is a case-control study comparing three groups, HIV-1 seropositive slow progressors (SP), HIV-1 seropositive rapid progressors (RP) and seronegative controls (CTR) [38]. We have previously published the exhaustive genotyping of SNPs from cytokines and cytokine receptors genes in that cohort [2,22]. In these works, we had computed the subhaplotypes derived from promoter SNPs by using the direct method and the comparison of the distribution of these subhaplotypes in the SP, RP, and CTR groups had led to the identification of a few genetic associations with AIDS progression. In the present study, we have recomputed these subhaplotypes with the use of the global method. We found that some positive signals (i.e. associations) found by the direct method have disappeared when using the global method (*IL4 Receptor *and *IL10 Receptor *[22]). On the contrary a test for association that seemed to be negative for the promoter of *IL6 *became significant [2]. All these results are summarized in Table 5.

**Table 5:** Modification of the results obtained in the GRIV case-control study when using the various subhaplotyping methods

**Genes**	**Sub-haplotype**	**p-value direct Rmax subhap**	**p-value global Rmax subhap**	**p-value Combination Rmax subhap**
IL10Receptor	Exon	0.026 A.H cases: 100% A.H controls: 100%	*0.103 A.H cases: 100% A.H controls: 100%	0.093 A.H cases: 88% A.H controls: 99%
IL4Receptor	Promoter	0.019 A.H cases: 100% A.H controls: 100%	*0.072 A.H cases: 100% A.H controls: 100%	*0.088 A.H cases: 100% A.H controls: 98%
IL6	Promoter	0.059 A.H cases: 100% A.H controls: 100%	0.012 A.H cases: 100% A.H controls: 100%	*0.009 A.H cases: 82% A.H controls: 90%

*Best method regarding the missing data level.

This table presents the p-values found for the Fisher's exact tests comparing the subhaplotypes distributions between seropositive patients of the GRIV cohort (cases) and seronegative subjects (controls). The subhaplotypes were computed either with the direct Rmax method as previously published [2, 43], or with the global Rmax method, or with the combination Rmax method described in our study. The percentage of missing information was respectively 6.7%, 11.1% and 14.8% for the *IL10R*, *IL4R*, and *IL6 *genotypic data. One can see that some signals which were previously published as positive (p < 0.05) using the direct method become negative, while some signals which were previously published as negative become much stronger thanks to the novel subhaplotyping methods. For *IL10R *we have a deficit of information for cases and as consequences a lower percentage of assigned haplotypes in the combination method which is more restrictive.

*A.H cases*: percentage of assigned haplotypes attributed in the tests for cases

*A.H control*: percentage of assigned haplotypes attributed in the tests for controls.

As the global method has very often a lower error rate, we conclude that the positive signals found in these studies were likely to be artifacts of the direct subhaplotyping while previously negative tests may have missed real associations in AIDS progression.

## Discussion and conclusion

In this study, we have confirmed that the error rate found in the resolutions determined by the best haplotyping software known to date, PHASE, could be non negligible even when there were no missing information in the genomic data [13,16,20,36]: it ranged from 1% to 6% according to the selection of SNPs (see Table 1 and 2). Errors were also observed at the level of the haplotypes frequencies (Table 1 and 2). In reality, when dealing with genotypic information obtained experimentally, there is often missing information and our study shows that in that case, the error rate for the estimation of haplotypes can jump even higher, reaching 25% in some instances (Table 1). This has led us to develop an alternative method to estimate haplotypes, the "global method". The rationale of the global method is to use the information contained in other SNPs, which are not used in the direct haplotyping, in order to limit the impact of missing data: for instance, the presence of linkage disequilibrium between SNPs might supplement missing data on certain SNPs.

We performed tests on genomic datasets for which haplotypes had been determined exactly through biological experimentation and also on simulated data. We generated randomly missing genotypic information in these datasets and computed partial haplotypes (subhaplotypes) from subsets of selected SNPs. We found that the global approach, which first computes the haplotypes from all the available SNPs and then extracts the subhaplotypes corresponding to the selected SNPs, reproducibly led to better estimations with significantly lower error rates (Tables 1 and 2).

Since biologists like to work with exact data, we also tried to work on the resolutions exhibiting a significant reliability as determined by PHASE: resolutions exhibiting a probability higher than 70% or higher than 50%. With this approach, the global method still yielded a lower error rate than the direct method (Table 3). It appears that when one increases the cut-off to assign a resolution the final error rate slightly diminishes while the number of patients being assigned a subhaplotype diminishes rather importantly (Table 3).

We finally tried to combine the global and direct methods for discordant patients (patients for which the haplotypes computed by the direct and global method were different). For that, we used the subhaplotype computed by the direct method if there was less missing information in the SNPs selected for subhaplotyping than in the remaining SNPs, or the global method in the opposite case. We found that this combination method could be a useful compromise when the level of missing information in the population was high: the relative individual error rate was smaller than that of the global method based on Rmax but some patients were not assigned an haplotype (Table 4).

The fact that the global method yields better results than the local method is not a surprise knowing the importance of linkage disequilibrium inside genetic loci. Indeed, Marchini et al. found similarly that for the computation of the r^2 ^coefficients it was more reliable to use large number of SNPs instead of pairwise comparisons [20].

In practice, if there is not too much missing information (less than 10%), the global method using the PHASE Rmax resolution works well with nearly all subjects being assigned a subhaplotype and with an error rate below 10% (Table 1 and 2). If there is more missing information (more than 10%), it might be interesting to use the combination method knowing that 90% of the subjects are assigned a subhaplotype among which less than 8% have a wrong haplotype (Table 4).

We have demonstrated the practical interest of this new subhaplotyping method in our GRIV genomic dataset: we had previously genotyped the cytokine and cytokine receptors in the GRIV cohort and we had estimated subhaplotypes of the promoter regions by direct subhaplotyping [2,22]. In the present work, we have recomputed the subhaplotypes of the promoter regions using the more precise SUBHAP software: we found that associations previously described for *IL4R*, *IL10R *subhaplotypes did not hold, while signals appeared much stronger for an *IL6 *subhaplotype (Table 5).

This work has extensively evaluated the impact of missing data on subhaplotyping and it emphasizes that the level of missing information in the genomic data is a critical issue: the practical impact is not negligible since in our experimental genotyping of the GRIV cohort, the rate of missing data may reach 20% for some SNPs. Such rates have also been widely described in the literature [39-41]. This work also underlines that the genetic structure of the SNPs in the population is an important issue since the error rates may vary from one population to the other (see Table 1 and 2) and it could certainly be interesting to take into account other parameters such as the LD and minor allele frequencies to help optimize the subhaplotyping procedure.

Current genomic studies, such as the Hapmap project, aim at minimizing the number of SNPs necessary to perform genetic associations in complex diseases by using tagSNPs. These studies do not consider the missing information problem inherent to any genotyping experiment which will often prevent the optimal haplotyping of the patients for disease genetic association studies. Our results suggest that if the Hapmap data are evidently very useful in targeting genetic regions of interest, an extensive genotyping with all SNPs in a sensitive region will however likely be needed to infer correct subhaplotypes.

In conclusion, the subhaplotyping method that we described here will allow to improve genetic association studies with complex diseases and take the best advantage of the available genotype data. The global and combination methods are available with Subhap software [42].

## Methods

### The GH1 haplotypes data set

This haplotypic data set was determined empirically by Haran et al. [34] from 154 patients who were recruited of the British army. The promoter of the growth hormone (*GH1*) gene spans 535 bps, and is very strongly polymorphic with 14 SNPs whose minor allele frequency (MAF) is greater than 1% in the studied population. By cloning and genotyping 154 patients [34], the authors managed to experimentally define 38 different haplotypes based on these 14 SNPs, including 18 haplotypes with a global frequency higher than to 1%. We excluded the only patient implicating a tri-allelic SNP to simplify the calculation: we thus only used 153 patients of this cohort.

The *GH1 *gene SNPs presents only one perfect LD and does not include any haploblock (Fig 1) which limits the skewing of the results and makes this genomic dataset more reliable for the comparison of the *direct *and *global *methods.

### The APOE haplotypes data set

This haplotypic dataset was determined experimentally by Orzack et al. [33] using a long-range allele-specific PCR on 80 unrelated individuals from 3 ethnic groups: 18 Asian, 19 African and 43 Caucasian individuals. The *APOE *locus is composed of 9 SNPs with MAF>1%. 17 haplotypes were identified experimentally. The level of LD between the *APOE *SNPs was also very low, as for *GH1 *polymorphisms. The *GH1 *and *APOE *data sets are very useful for our goal. Indeed, if we show that the global method is more efficient on them, that advantage will be even stronger for common datasets because they generally exhibit more LD.

### The simulated haplotypes data set

This haplotypic data was created with COSI package developed by Schaffner et al. [35] based on a coalescent model. We have generated 10 data sets of 30 SNPs on 100 unrelated individuals simulated with constant recombination rate across the region, constant population size, and random mating.

### The GRIV data sets

The GRIV cohort is composed of 400 Caucasian HIV-1 positive patients with extreme profiles of progression to AIDS (Slow Progression or Rapid Progression) and has been extensively genotyped by PCR-sequencing on various genes of the immune system [38]. In addition, 400 healthy subjects of similar ethnic origin were also genotyped as controls (CTR). In the present study, we have used the genotypes obtained on genes analyzed in the cohort and previously reported: cytokines and their receptors [2,43]. Unlike the *GH1 *and *APOE *data sets previously described, we do not dispose of the real haplotypes for this population.

### Creation of missing data

The data set from the *GH1 *study was a complete data set. In order to study the influence of missing data on the accuracy of the results, missing data were artificially generated inside the *GH1 *data set. To be more realistic, missing data was distributed randomly across genomic datasets. We applied similar levels of missing data to the *GH1*, *APOE *and *simulated *datasets: 2%, 5%, 10%, 15% and 20%.

### Haplotyping software

We have chosen to use the PHASE software [13,19] to infer haplotypes. Indeed, many studies some of which performed on the *GH1 *datasets have compared the different haplotyping algorithms and came to the conclusion that the PHASE algorithm performed better [16, 17, 44, 45] with a lower error rate and a higher number of solved patients. PHASE is based on a Markov chain of Monte Carlo with a recombination model based on the decay of LD with distance. The PHASE parameters were optimized using the empirical haplotypes of the *GH1 *promoter: the thinning interval (steps through the Markov chain per iteration) and the number of runs (of the algorithm) didn't seem to alter significantly the results. The number of iterations on these data which apparently yielded the lowest error rate and the best number of inferred haplotypes was 100 iterations and 1000 burn-in (100, 500 and 10000 iterations were tested). The other parameters were set by default.

Subjects with more than 50% missing information were removed in order to avoid estimating haplotypes when there was too much data lacking.

### Subhaplotyping methods tested

#### The direct method

A subset containing only the genotypes of the selected SNPs was extracted from the whole data set for all the individuals. The haplotypes for these SNPs were then inferred by haplotyping this data set with the PHASE software.

#### The global method

The haplotypes were first inferred with the PHASE algorithm from the whole data set containing all the SNPs genotypes in each gene. This initial haplotyping provides for each patient the diplotype derived from all the SNPs and encompasses automatically the SNPs selected for subhaplotyping. The subhaplotypes corresponding to the selected SNPs could then be extracted directly from this global data set, forming the subhaplotype data set.

#### The combination method

When the two methods disagree on the resolution for one patient, a resolution was chosen after assessing which method was the most reliable.

In the case of the combination method based on the Rmax resolution, the choice of the resolution depended on the rate of missing information in the SNPs used to estimate the subhaplotype. If the missing information was higher than 30% both in SNPs composing the subhaplotype and in the remaining ones used for the global method, we considered that the patient's haplotypes could not be solved.

### Comparison of the resolutions found by each method with the real subhaplotypes

The results obtained when using each subhaplotyping method were compared to the real subhaplotypes as determined experimentally. This comparison was done by using various coefficients measuring the error rate that are described in the paragraphs hereafter. We have used the ANOVA model to test if there was any statistical difference between the error rates obtained from the two methods.

#### IER and SimER: error rates for haplotype assignments

The resolution rate (Res Rate) is the proportion of individuals for which a diplotype was found by the subhaplotyping method. Res Rate thus ranges from 0 to a maximum of 1 when all patients are assigned an haplotype.

The individual error rate (IER) is the proportion of subjects whose inferred diplotype is not correct. In case the Res Rate was <1, we called relative IER (Rel IER) the proportion of subjects whose inferred diplotype is not correct among all the subjects who were assigned a diplotype. In case the Res Rate was <1, we called absolute IER (Abs IER) the proportion of subjects whose inferred diplotype was not correct among the whole population.

The similarity error rate (SimER) is another measure of similarity between the estimated haplotypes and real haplotypes, which was developed by Stephens et al. [13]: it is based on the percentage of errors found at the level of SNPs for each haplotype.

#### I_F_: error rate for the frequencies of the attributed haplotypes

*I*_*F *_[37] measure how closely the inferred and empirical haplotype frequencies correspond and is given by:

IF=1−12∑k=1h|pek−ptk|
 MathType@MTEF@5@5@+=feaafiart1ev1aqatCvAUfKttLearuWrP9MDH5MBPbIqV92AaeXatLxBI9gBaebbnrfifHhDYfgasaacH8akY=wiFfYdH8Gipec8Eeeu0xXdbba9frFj0=OqFfea0dXdd9vqai=hGuQ8kuc9pgc9s8qqaq=dirpe0xb9q8qiLsFr0=vr0=vr0dc8meaabaqaciaacaGaaeqabaqabeGadaaakeaacqWGjbqsdaWgaaWcbaGaemOrayeabeaakiabg2da9iabigdaXiabgkHiTmaalaaabaGaeGymaedabaGaeGOmaidaamaaqahabaWaaqqaaeaacqWGWbaCdaWgaaWcbaGaemyzauMaem4AaSgabeaakiabgkHiTmaaeiaabaGaemiCaa3aaSbaaSqaaiabdsha0jabdUgaRbqabaaakiaawIa7aaGaay5bSdaaleaacqWGRbWAcqGH9aqpcqaIXaqmaeaacqWGObaAa0GaeyyeIuoaaaa@47AF@

where *p*_*ek *_and *p*_*tk *_are the inferred and empirically determined frequencies for the k*th *haplotype, and *h *is the number of haplotypes. *I*_*F *_range from 0 to a maximum value of 1 when the frequencies match perfectly

## Authors' contributions

CC and OD have worked on developing the methods and programs used in this study under the direct supervision of JFZ who conceived the study. RA, MG and JLS worked on evaluating the accuracy of PHASE in the tested datasets. HD provided the real GRIV genotypic data. All authors read and approved the final manuscript.
